# WT1 expression in vessels varies with histopathological grade in tumour-bearing and control tissue from patients with breast cancer

**DOI:** 10.1038/s41416-018-0317-1

**Published:** 2018-10-30

**Authors:** Richard J. McGregor, You-Ying Chau, Timothy J. Kendall, Mara Artibani, Nicholas Hastie, Patrick W. F. Hadoke

**Affiliations:** 1 University/BHF Centre for Cardiovascular Science, Edinburgh, UK; 2 MRC HGU at the MRC Institute of Genetics and Molecular Medicine (IGMM), Edinburgh, UK; 3 Division of Pathology, University of Edinburgh, Edinburgh, UK; 4 Weatherall Institute of Molecular Medicine, University of Oxford, Oxford, UK

**Keywords:** Breast cancer, Tumour angiogenesis, Cancer genetics, Gene expression, Gene regulation

## Abstract

**Background:**

The Wilms’ tumour protein (WT1), which influences tumour development and angiogenesis, is a promising therapeutic target in breast cancer. We hypothesised that WT1 expression would vary in endothelial cells in distinct sub-classifications of breast cancer.

**Methods:**

WT1 expression and vascular density were quantified by immunohistochemical analysis of human (*n* = 57) and murine breast cancers. Human tumours were sub-classified by histopathological grade, ER status and HER2 enrichment.

**Results:**

WT1 was identified in endothelial (and epithelial and smooth muscle) cells in tumours and tumour-free tissues (controls) from patients and mice with breast cancer. WT1 expression was higher in tumours than in controls, but this was not due to increased endothelial WT1. Vascular WT1 in cancers decreased as histopathological grade increased. WT1 was higher in ER-positive versus ER-negative cancers. Strikingly, reduced WT1 expression in controls correlated with an increased Nottingham Prognostic Index score.

**Conclusions:**

Expression of WT1 is increased in breast cancers but this is not limited to the vascular compartment. The association between reduced WT1 in tumour-free tissue and poor prognosis suggests a protective role for WT1 in the healthy breast.

## Introduction

Breast cancer, the most common female malignancy across the developed. and developing world, is the primary cause of death among women globally.^1^ Recently, the Wilms tumour protein (WT1) has emerged as a potential therapeutic target in this condition.

*WT1*, a multi-functional gene fundamental to mammalian embryological development,^2–4^ is normally only expressed in discrete sites in the adult (e.g. in the mesothelium surrounding the visceral organs, the glomerular podocytes of the kidney, the sertoli/granulosa cells of the testis/ovary and 1% of bone marrow cells).^5–10^ However, substantial evidence, both from solid tumours and leukaemia, suggests a role for *WT1* as an oncogene.^11–15^ Indeed, such evidence linking WT1 expression with tumour formation led to the United States National Cancer Institute ranking WT1 as the cancer antigen with greatest potential as a target for immunotherapeutic agents.^16^ Furthermore, since WT1 expression in the vascular endothelium is implicated in the regulation of angiogenesis,^17^ increased expression of WT1 in endothelial cells may contribute to tumour formation, for example Wagner et al.^18^ reported *WT1* expression in endothelial cells in 95% of 113 solid (lung, ovarian, pancreatic, breast and bladder) tumours, yet *WT1* was not expressed in adjacent healthy tissue. Crucially, conditional knockout of *Wt1* from endothelial and *Tie2*-expressing cells markedly reduced both vascularisation and growth of melanoma (B16) and lung carcinoma (LLC1) in murine xenografts.^19^

Considerable evidence has implicated *WT1* in the pathogenesis of breast cancer.^20–25^ High *WT1* mRNA levels in breast tumours were associated with a lower 5-year disease-free survival rate.^26–28^ In addition, immunohistochemical analysis associated cytoplasmic WT1 expression in invading tumour cells with a more biologically aggressive phenotype (e.g. oestrogen receptor (ER)-negative tumours > 2 cm in size).^22,25^ However, the biological basis behind WT1 expression and poor clinical outcome is not well understood.^29^ This may be due to inconsistencies in the published data regarding WT1 mRNA,^27,30^ the protein expression levels in breast tumours^31,32^ or the numerous WT1 isoforms that may have divergent functional roles^5,33^^,^^3,20^. Indeed, recent work suggests that the truncated WT1 transcript starting from intron 5 is tumour specific.^30^ Furthermore, few studies have assessed WT1 expression in histopathological sub-types of breast carcinoma. This may be important as breast cancer is a profoundly heterogeneous disease whose correct classification is essential for optimal management.^34^ Traditionally, prognosis was determined using a series of conventional markers, including tumour size, lymph node involvement, histological grade, oestrogen receptor (ER) status and epidermal growth factor receptor-2 (HER2) amplification status.^35^ More recently, improved analysis of gene expression has challenged the conventional view that breast cancer is a single disease.^36,37^ This may be highly significant when contemplating peptide-based cancer immunotherapies targeting WT1.^38^

Clearly, a better understanding of the relationship between WT1 and breast cancer is required to inform the development of immunotherapy for targeting WT-positive tumour cells in this condition. This investigation addressed the hypothesis that WT1 expression is increased in endothelial cells in human breast cancers. The specific aims were to determine whether: (1) WT1 expression is increased in vascular endothelial cells in human breast cancers; (2) expression of WT1 varies according to Grade and histopathological stratification of tumours; and, (3) whether a mouse model can be used to assess the role of WT1 in breast cancer.

## Materials and methods

### Tissue collection and histopathological analysis of human breast cancer

All human cancers, and matched healthy control tissue from the same patients, were obtained via the NRS BioResource and Tissue Governance Unit funded by the Chief Scientists Office (CSO) with Research Ethics Committee approval (15/ES/0094). Samples were handled in accordance with the approved guidelines and written informed consent was obtained from all subjects.

Sections (4 µm) were taken from formalin-fixed, paraffin-embedded tumour blocks from 60 cases of female human carcinoma of no special type (ductal NST), and matched non-lesional breast tissue. Consecutive cases were selected where block keys in anonymised reports allowed identification of blocks from a random, and unknown, period between 2010 and 2013. Equal numbers of Grade I–Grade III tumours from the right and left breast were selected. The histopathological grade, ER status, progesterone receptor status, maximum tumour dimension (mm) and HER2 enrichment status were obtained from the anonymised NHS pathology report. Haematoxylin and eosin (H&E) stained sections from each case were reviewed by a Consultant Pathologist to confirm appropriate block selection.

### Immunoperoxidase staining

Twelve representative tumour samples (x4 Grade I, x4 Grade II, x4 Grade III) and matched controls (*n* = 12), were stained for WT1 (1:500, C19, #SC- 192, Santa Cruz, USA). All samples were visualised using the Provis AX-70 Optical Microscope with Axiocam HRc Camera.

### Immunofluorescence

A total of 120 tissue sections (60 tumours and 60 matched controls) were stained for WT1 (1:500, C19, SC- 192, Santa Cruz, USA) and CD31 (1:200, Ab28364, Abcam®, UK). For each sample, five 200 μm × 200μm regions of interest were randomly selected and analysed using confocal microscopy to quantify the mean number of: (a) CD31-expressing cells (endothelial cells); (b) all WT1 expressing cells; (c) cells co-expressing WT1/CD31; (d) vessels; and (e) WT1^+^ vessels (defined as a vessel with 2 or more WT1^+^ endothelial cells). The percentage of WT1^+^ vessels for each region of interest within a section was then calculated. These 5 percentages were used to create a mean value for each section. All analyses were performed blind using an LSM 710 Confocal microscope (Carl Zeiss, Germany). In addition, the WT1 polyclonal antibody was combined with its matched blocking peptide (1:500, SC-192P, Santa Cruz, USA) in a direct competition test on three patient samples (both lesional and non-lesional control). Finally, an IgG isotype control (1:100, Ab172730, Abcam®, UK) was used on human breast tissue sections from lesional (*n* = 3) and non-lesional controls (*n* = 3) to rule out non-specific cell surface staining of the WT1 antibody. A detailed analysis of the use of the C19 WT1 polyclonal antibody in a variety of human cancers has been described previously.^39,40^

### Preclinical murine model of breast carcinogenesis

The preclinical murine *C3(1)/Tag* breast cancer line,^41^ was used to examine murine breast carcinomas, with standard Friend Virus B-type (FVB) females acting as controls. All animal experiments were performed in compliance with the UK Animals (Scientific Procedures) Act 1986, under Project Licence PPL 60/3788 approved by the UK Home Office. Individual experiments were approved by the local University of Edinburgh Ethical Review Committee.

### Histopathological analysis of the C3(1)Tag murine model of breast cancer

Formalin-fixed, paraffin-embedded blocks were prepared after retrieval of the tumour and healthy control breast tissue from the respective mice. Sections (4 µm) were stained with H&E then reviewed and verified by a Veterinary Surgeon and Member of the European College of Veterinary Pathologists, according to the consensus report and recommendations into the mammary pathology of genetically engineered mice, Annapolis 1999.^42^ Six tumours (*n* = 6) and healthy control samples (*n* = 6) were stained for Wt1 (1:500, C19, SC-192, Santa Cruz, USA) and CD31 (1:200, Ab28364, Abcam®, UK) using double immunofluorescence with tyramide signal amplification—utilising a protocol previously published by our group.^43–45^ For each sample, five randomly selected, 200 μm × 200 μm regions of interest were analysed using confocal microscopy allowing quantification of the mean number of: (a) CD31-expressing cells; (b) Wt1 expressing cells; (c) cells co-expressing Wt1/CD31; (d) vessels; and (e) Wt1^+^ vessels (defined as a vessel with 2 or more Wt1^+^ endothelial cells). All analyses were performed blinded to genotype using an LSM 710 Confocal microscope (Carl Zeiss, Germany).

A single kidney from each animal was harvested and processed for Wt1 immunofluorescence as a positive control. In addition, the WT1 polyclonal antibody was combined with its matched blocking peptide (1:500, SC-192P, Santa Cruz, USA) in a direct competition test on three kidney samples as per the manufacturer’s instructions. Finally, an IgG isotype control (1:100, Ab172730, Abcam®, UK) was used on kidney sections (*n* = 3) of FVB control animals to rule out non-specific cell surface staining of the WT1 antibody.

### Statistics and data analysis

Parametric data are expressed as mean (±standard deviation), whilst non-parametric data are expressed as median (±interquartile range). In all unpaired analyses Student’s *t*- and Mann–Whitney *U* tests were used for parametric and non-parametric data, respectively. A one-way analysis of variance (ANOVA) (plus Tukey’s multiple comparison test) and Kruskal–Wallis Test with Dunn’s multiple comparisons were used when comparing the means of two or more unpaired samples from parametric and non-parametric data samples, respectively. Correlations were calculated using a two-tailed Pearson’s test. Statistical significance was defined as *p* < 0.05. All analyses were performed using Prism 6 for Mac OS X (GraphPad Software Inc., USA).

## Results

### Histopathological analysis of human breast cancer

All 60 human tumour samples and matched controls were assessed independently by a Consultant Pathologist prior to analysis. Two tumour cases were excluded as no tumour was identified in the selected block. The histopathological grade of the 58 remaining tumour samples documented in the original report was confirmed. Ten non-lesional control blocks were excluded from analysis as they contained tumour.

### Identification of WT1 in human breast cancer and matched-control tissues

In a selection of healthy breast tissue samples (*n* = 12) immunohistochemical staining identified WT1 in some of the endothelial cells lining the arteries, veins and capillaries (Suppl. Fig. 1). Moreover, some smooth muscle cells of the arterial wall, and epithelial cells of the terminal duct lobular unit (TDLU) were also WT1-positive. In matched breast cancer samples (*n* = 12; ×4 Grade I, ×4 Grade II, ×4 Grade III) WT1 staining was observed in tumour stromal cells, epithelial cells of adjacent benign TDLU, smooth muscle cells and endothelial cells lining the arteries, veins, and capillaries of all tumour grades (Suppl. Fig. 1).

More detailed analysis of WT1 expression in breast cancer samples and matched controls was performed using double Immunofluorescence. Of the 58 tumours obtained, 1 (and its matched control) was excluded as the patient had received pre-operative chemotherapy. Consequently, 57 tumour samples and 49 controls were analysed following double immunofluorescence.

**Fig. 1:**
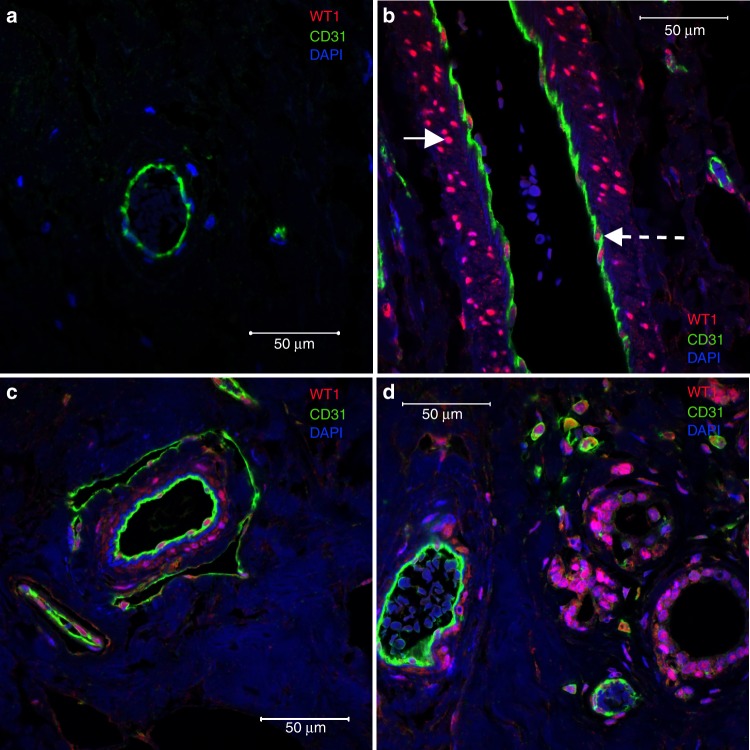
WT1 is expressed in human breast cancer and in healthy control tissue. Double immunofluorescence showing **a** healthy breast tissue displaying no endothelial WT1 expression in a small capillary. **b** Healthy breast tissue exhibiting clear expression of WT1 in endothelial (red) (denoted by broken white arrow) and smooth muscle cells (red) (denoted by white arrow) in the arterial wall. **c** Breast cancer (Grade II, ER-positive, HER2-negative) sample displaying WT1^+^ (red) endothelial cells (CD31^+^; green) in both the artery and small capillaries; with WT1-positive smooth muscle cells evident in the arterial media. **d** Breast cancer (Grade III, ER-positive, HER2-positive) sample exhibiting WT1^+^ (red) ducts, arteries and tumour stromal cells. Wt1 (red), CD31 (green), DAPI (blue). Scale bars represent 50 µm

WT1 immunopositivity was identified in several cell types both in control breast tissue and in breast cancers (Fig. 1). In control breast tissue, vessels (arteries, veins and capillaries) could be identified with no WT1 staining of the endothelium (Fig. 1a) or underlying smooth muscle. In contrast, some vessels in these tissues contained a proportion of WT1-positive endothelial and smooth muscle cells (Fig. 1b). Breast cancer samples also exhibited a combination of vessels that did not express WT1, alongside those containing WT1-positive (endothelial and smooth muscle) cells (Fig. 1c, d). In addition, epithelial cells lining the TDLU in health and disease, alongside the tumour stromal cells, were also positive for WT1 (Fig. 1d).

**Fig. 2:**
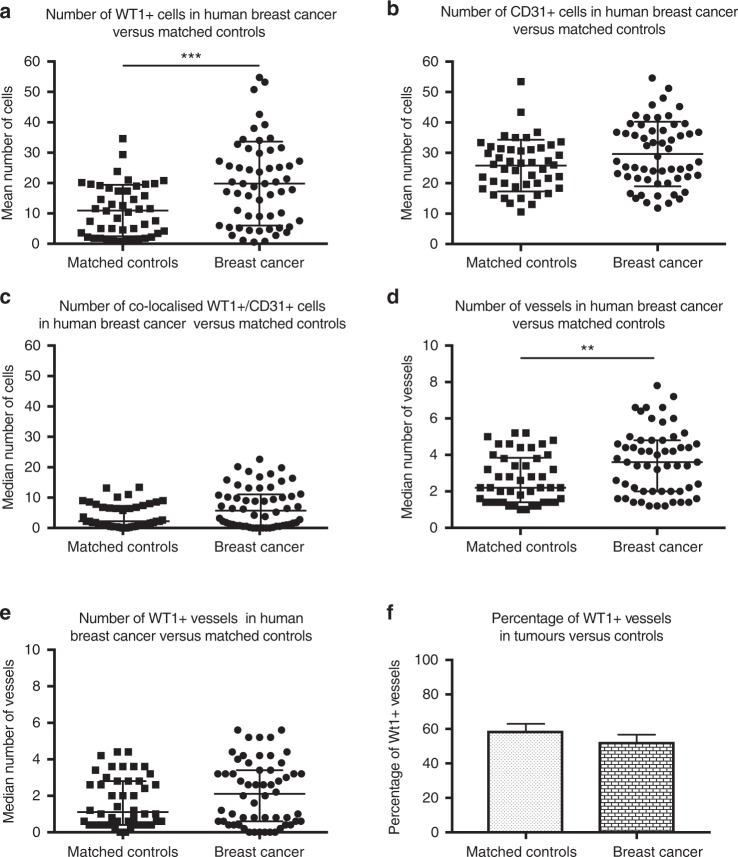
WT1 expression is increased in human breast cancers compared with matched-control tissue. The number of WT1^+^ cells was higher in tumours than in matched-control tissue (**a**). No difference was detected in the number of CD31-expressing cells (**b**) or the number of cells that co-expressed WT1 and CD31 in the tumours versus matched controls (**c**). The number of vessels was higher in tumours than in controls (**d**) but no difference was detected in the number of WT1 expressing vessels between the tumours and matched-control tissue (**e**). The percentage of WT1-positive vessels was not different in tumours and controls (**f**). **a**, **b** Data are mean ± standard deviation where ***p < 0.0005 by unpaired Student’s *t*-test, **c**–**e** data are median ± interquartile range where **p < 0.01 by Mann–Whitney *U* test (*n* = 55)

Quantification demonstrated that the total number of WT1-positive cells was higher in the human breast cancer samples (*n* = 57) than in matched controls (Fig. 2a). There was no difference detected in the number of CD31-positive cells (*p* = 0.053) (Fig. 2b) or the number of cells that co-expressed WT1/CD31 (Fig. 2c). The total number of vessels was higher in breast cancers than in control tissue (Fig. 2d), but no difference was observed in the number (Fig. 2e) or percentage (Fig. 2f) of WT1-positive vessels.

### WT1 expression decreases with histopathological grade of breast tumour

**Fig. 3:**
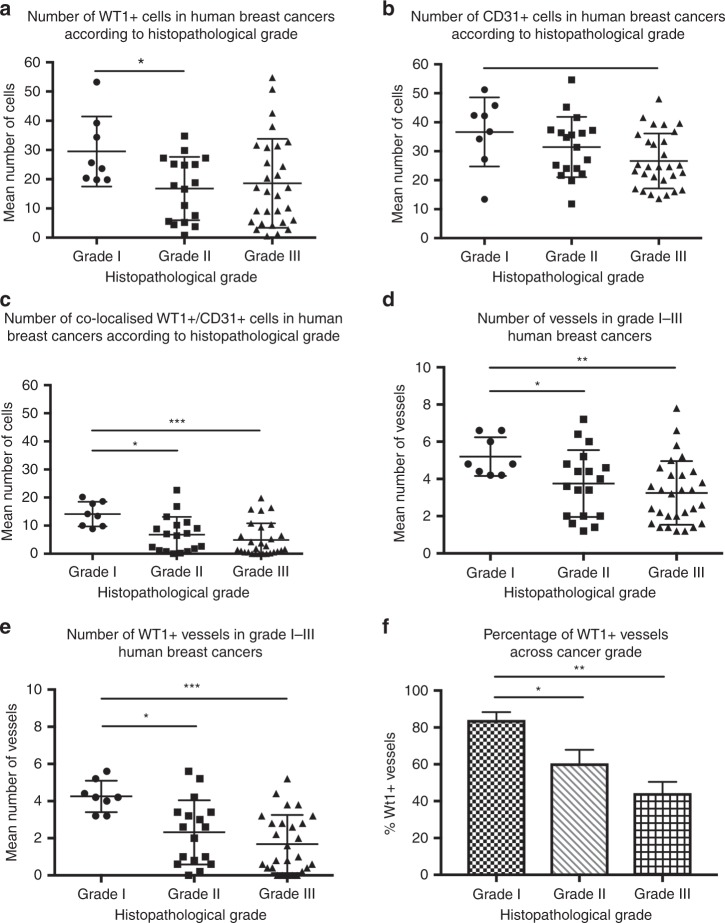
WT1 expression drops as histopathological grade increases in human breast cancers. Grade I tumours expressed more WT1^+^ cells than Grade II cancers, but no difference was otherwise detected between tumour Grades (**a**). Grade I tumours expressed more CD31-positive cells than with Grade III cancers (**b**), alongside more cells that co-expressed WT1 and CD31 when compared to both Grade II and Grade III tumours (**c**). The number of vessels was higher in Grade I versus Grade III tumours (**d**) and there were more WT1-positive vessels in Grade I tumours compared with both Grade II and Grade III tumours (**e**). The percentage of WT1-positive vessels within the tumours appeared to decrease as histopathological Grade increased, and this was significant when Grade I cancers were compared with Grade III (**f**). Data are mean ± standard (where n = 8 Grade I, 17 Grade II, 29 Grade III, deviation); (*p < 0.05, **p < 0.01 and ***p < 0.005 by unpaired Student’s *t*-test)

Of the 56 (from the total of 57) human tumours with grade available, there were 8 Grade I, 17 Grade II, and 29 Grade III tumours analysed following immunofluorescent staining. Analysis of these data demonstrated that WT1 expression decreased with rising histopathological Grade (Fig. 3). The total numbers of WT1-positive cells (Fig. 3a), CD31-positive cells (Fig. 3b), and WT1/CD31 dual-positive cells (Fig. 3c) were all higher in Grade I tumours. In addition, the numbers of vessels (Fig. 3d), and WT1-positive vessels (Fig. 3e) were also higher in Grade I breast cancers compared to Grade II tumours. Interestingly, the percentage of WT1-positive vessels within the tumours also decreased as tumour grade increased (Fig. 3f).

### WT1 expression in cancer sub-types versus matched-control tissue

**Fig. 4:**
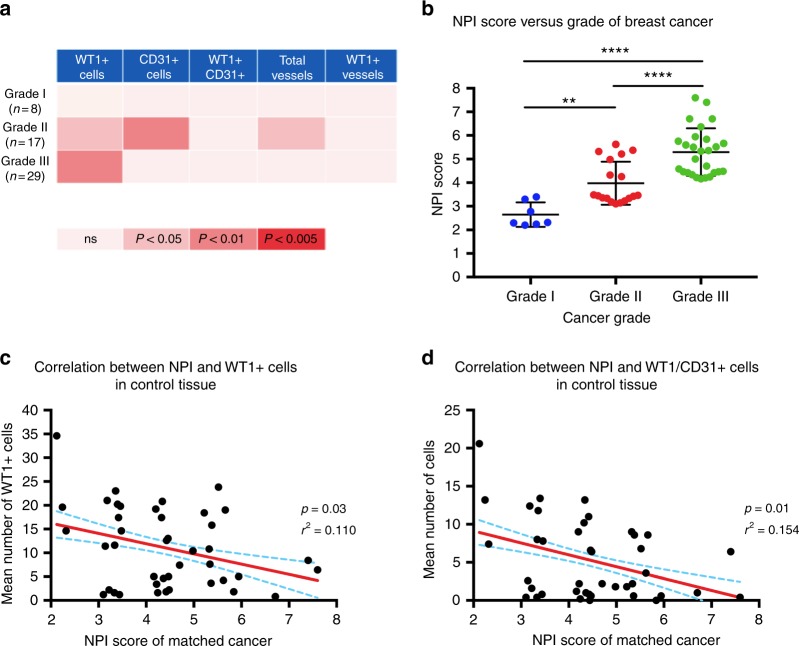
Relative WT1 expression in tumours increases with Grade as a result of reduced WT1 expression in matched-control tissues. **a** Heat map depicting the variation in expression levels of WT1 across Grade I–III cancers relative to match control tissue. Statistically significant results are represented by the degree of colour change within the panels. For example, no difference was detected between Grade I tumours and their matched controls across any of the domains (denoted by the neutral colour). By contrast, Grade III cancers exhibited increased numbers of Wt1+ cells versus controls (denoted by red). **b** The Nottingham Prognostic Index (NPI) Score increase with Grade (one-way ANOVA, where *p < 0.01 and ****p < 0.0001) for Grade I tumours. Linear regression analysis revealed that a decrease in the numbers of WT1-positive cells (**c**), and of cells that co-express WT1 and CD31 (**d**), in the matched-control breast tissue from patients with breast cancer is associated with an increase in the NPI score for those patients; *n* = 26 Grade III cancers, **c**, **d** parametric data analysed with Pearson’s correlation analysis

Summarising the differences in cellular and vascular expression of WT1 (Fig. 4a) demonstrated that Grade II and III, but notably not Grade I, breast cancers exhibited greater expression of WT1-positive cells than matched-control tissue.

### WT1 expression and the Nottingham Prognostic Index (NPI) score

The NPI was calculated using the standard criteria^46^ for 50 out of the 57 cancers (not all had their nodal status available). According to the scoring system, NPI < 3.4 equates to an 85% 5-year survival, versus NPI > 5.4, which corresponds to a 5-year survival of <50%.^47^ Stratification of cancers by Grade against NPI score demonstrated a clear increase in NPI with increasing tumour Grade (Fig. 4b).

In the matched-control breast tissue from patients with cancer, linear regression analysis revealed that the NPI increases as the number of WT1-positive cells decreases (Fig. 4c). A similar pattern was observed with cells that co-expressed WT1 and CD31 (Fig. 4d). This therefore suggests that WT1 expression in health, may be protective. No difference was detected in the relationship of WT1^+^ cells, WT1/CD31 co-expression, or the percentage of WT1^+^ vessels, with the NPI score, when the cancers were stratified by Grade (not shown).

### WT1 expression and HER2 status

The association between WT1 and HER2 status was assessed in 57 cancers. Statistical analysis was undertaken to ascertain whether the expression pattern of WT1 differed between the HER2-positive (*n* = 17) and HER2-negative cancers (*n* = 40). No difference was detected between tumour sub-types in the number of: WT1-positive cells; CD31-positive cells; WT1/CD31-positive cells; blood vessels; or WT1-positive blood vessels (Suppl. Fig. 2).

### WT1 expression in triple-negative breast cancers

There were 15 triple-negative breast cancers in this cohort, with 14 matched controls. The total number of WT1^+^ cells was increased in the triple-negative cancers versus tumour-free sections from matched controls. There was no difference detected in the total number of CD31^+^ cells, WT1/CD31 co-localisation, number of vessels, number of WT1^+^ vessels, or the percentage of WT1^+^ vessels (Suppl. Fig. 3).

### WT1 expression and oestrogen receptor status

**Fig. 5:**
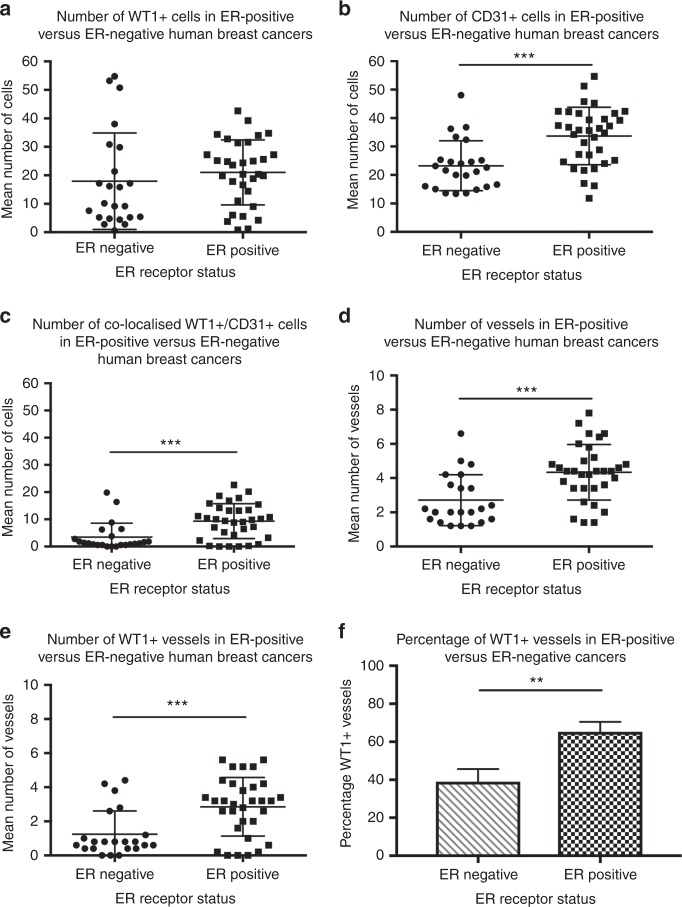
The relationship between WT1 expression and oestrogen receptor (ER) status in human breast cancers. There was no difference between the number of WT1+ cells in ER-positive versus ER-negative breast cancers (**a**). ER-positive tumours had greater CD31 expression (**b**), WT1/CD31 co-localisation (**c**), total number of vessels (**d**), and number of vessels that were WT1-positive (**e**), when compared with their ER-negative counterparts. In addition, the percentage of WT1-positive vessels within the tumours was significantly greater in ER-positive than in ER-negative cancers (**f**). Data are mean ± standard deviation (*n* = 23 ER-negative tumours, *n* = 32 ER-positive tumours) ***p < 0.001 by unpaired Student’s *t*-test

ER status was available for 55 cancers; these cases were analysed to ascertain whether any difference in WT1 expression could be detected between ER-positive (*n* = 32) and ER-negative tumours (*n* = 23) (Fig. 5). The number of cells expressing WT1 was not different in ER-positive and ER-negative tumours (Fig. 5a). However, ER-positive tumours had a higher number of CD31-positive cells (Fig. 5b), WT1/CD31 co-expressing cells (Fig. 5c), total blood vessels (Fig. 5d), and WT1-positive blood vessels (whether expressed as total number (Fig. 5e) or percentage (Fig. 5f)).

### Comparison of WT1 expression in control tissues and breast cancers stratified by histopathological status

Supplementary Figure 4A depicts the differences in cellular and vascular expression of WT1 between a variety of breast cancer sub-types. Interestingly, all breast cancers, with the exception of the ER-negative tumours, expressed higher levels of WT1 versus that of the matched controls, whilst ER-positive cancers showed the most marked difference from controls, with higher of WT1-positive, CD31-positive, and WT1/CD31 double-positive cells, as well as higher numbers of both total and WT1-positive vessels (Suppl. Figure 5).

Stratification of cancers by histopathological status against NPI score (Suppl. Figure 4B) demonstrated that the majority of tumour types had a similar NPI scores (the exception being Grade I tumours). It is notable, for example, that the NPI scores of ER-positive tumours (which have relatively high expression of WT1 compared to controls) showed no significant different to those that were ER-negative (which had similar WT1 expression to control tissues).

### WT1 expression and vessel number are increased in the C3(1)Tag murine model of breast cancer

**Fig. 6:**
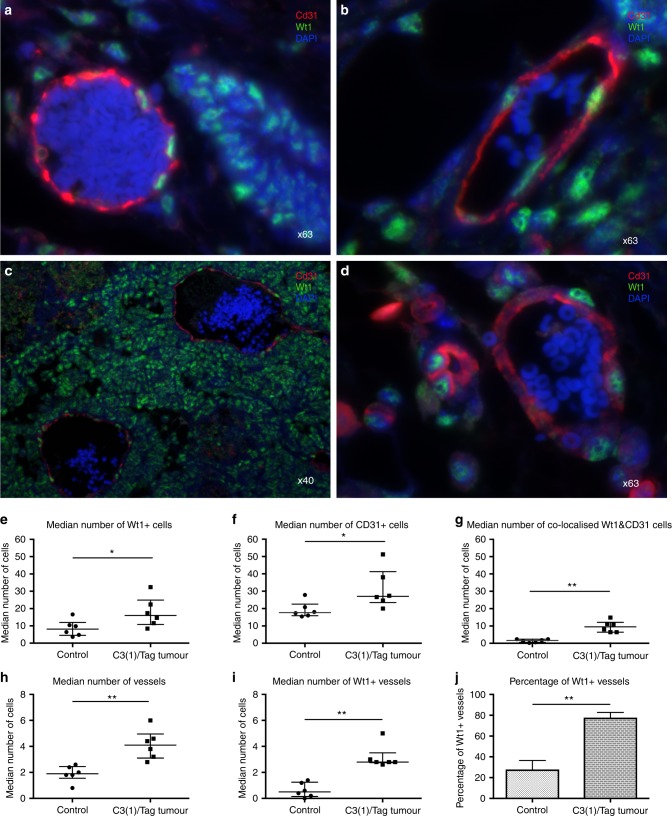
Double immunofluorescence showing Wt1 expression in the breast tissue of C3(1)/Tag mammary tumour mice versus Friend Virus B-type (FVB) controls. **a** Some epithelial cells of the terminal duct lobular unit (TDLU) were Wt1-positive (green) in the healthy FVB mouse. **b** A blood vessel within the mammary tissue of the control FVB mouse-positive for the endothelial cell marker, CD31 (red) and co-localising with Wt1 (green). **c** The tumour stroma of the C3(1)/Tag mammary carcinoma model exhibits global Wt1 (green) expression, whilst **d** depicts Wt1-positive (green) endothelial cells (red) at higher magnification in the same animals (note DAPI = Blue in all images). Compared with controls, tumours from the C3(1)/Tag mice exhibited higher numbers of: **e** Wt1-positive cells, **f** CD31-positive cells, **g** Wt1/CD31 double-positive cells, **h** total blood vessels, and **i** Wt1-positive blood vessels. **j** The percentage of Wt1-positive blood vessels was also increased in the C3(1)/Tag tumour model when compared to controls. Data are expressed as median ± interquartile range, where *p < 0.05 and **p < 0.01 by Mann–Whitney *U* test (*n* = 6)

In healthy murine breast tissue, a proportion of the epithelial cells of the TDLU expressed Wt1 (Fig. 6a). As in the human tissues, double immunofluorescence for Wt1 and CD31 of the mammary tissue of all six FVB controls revealed that a proportion of endothelial cells of the arteries, veins and capillaries expressed Wt1 (Fig. 6b). In samples from C3(1)Tag mice, intra-tumoral stromal cells were Wt1-positive (Fig. 6c), as were some endothelial cells of the arteries, veins and capillaries (Fig. 6d). TDLU epithelial cells within the tumours were also noted to be Wt1-positive (not shown). The numbers of Wt1-positive cells (Fig. 6e), CD31-positive cells (Fig. 6f), Wt1/CD31 double-positive cells (Fig. 6g), vessels (Fig. 6h) and Wt1-positive vessels (Fig. 6i) were all higher in tumours than in controls. In addition, the percentage of vessels expressing Wt1 was greater in the tumours than control tissue of FVB mice (Fig. 6j).

## Discussion

This investigation addressed the hypothesis that WT1 expression is increased in vascular endothelial cells in human breast cancers. Whilst it was demonstrated that global WT1 expression was elevated in cancer samples, this increase could not be attributed to increased expression in vascular endothelial cells (despite increased vascular density in these specimens). However, sub-analyses based on stratification of ER status demonstrated a higher WT1 expression in ER-positive than ER-negative cancers. Furthermore, a negative association was identified between WT1 expression and histopathological Grade. The observation that reduced cellular expression of WT1 in control tissue was associated with a worse NPI may suggest a protective role for WT1 expression in the healthy breast. Ideally, this investigation would have included control tissue from appropriately matched cancer free patients, but such samples were not available. Future studies should consider including disease-free control groups although acquiring tissue from matched, healthy controls will be challenging.

The results obtained show clearly that WT1 is expressed in the endothelial cells, and also in smooth muscle and epithelial cells, of tumour-free mammary tissue from patients with breast cancer. The pattern of expression was notable with only a proportion of cells expressing WT1 (e.g. a single vessel could contain only one WT1-positive endothelial cell). This identification of WT1 in the tumour-free sample is perhaps unsurprising, as human reproductive tissues undergo cyclic remodelling, with angiogenesis a key part of this physiological process.^48^ Whilst these results contrast with previous work reporting no WT1 expression in healthy tissue from patients with cancer,^18^ others have detected WT1 in tumour-free human mammary tissue.^22,25^ Interestingly, a recent study demonstrated that normal mammary tissue from a group (*n* = 5) of healthy donors expressed higher WT1 mRNA levels than tumour samples but lacked specific (exon 1A and intron 5) isoforms.^30^ It was also notable that a similar pattern of WT1 expression was seen in control tissue from the *C3(1)-Tag* transgenic mouse murine model of breast cancer. These data suggest that WT1, in several different cellular compartments, has a role in regeneration of breast tissue. Moreover, the finding that loss of WT1 and endothelial WT1 expression in control tissues is associated with poorer clinical outcomes (i.e. increasing NPI) is consistent with the homoeostatic role the gene has in other tissues and organ systems^30,47^. Future studies should determine the pattern of WT1 isoform expression in sub-types of human breast cancer. Unfortunately, molecular analysis was not possible in the current investigation as tissue extraction did not generate mRNA of sufficient quality for qPCR.

The demonstration that WT1 expression is increased in cancerous tissue compared with control sections from the same patients is consistent with previous reports.^19^ However, the mechanism underlying, and the role and significance of, this increase in WT1 remains poorly understood. For example, WT1 expression in invasive ductal carcinoma has been linked to both improved^49^ and poor patient outcomes.^26^ These conflicting findings may be due to the fact that analysing cancers *en masse* is too blunt an instrument, i.e. subtleties in expression levels between cancer sub-types may better inform our understandings of pathogenesis and the appropriateness of targeted therapies.

In our study, we found no correlation between WT1 expression and NPI score. However, in a sub-group of carcinomas with a particularly poor prognosis—triple-negative breast cancers—WT1 expression levels were significantly higher than in control tissue. This is consistent with published data that equates high levels of methylation across the WT1 gene in triple-negative breast cancers with elevated levels of expression and poor survival.^50,51^ It should be reiterated, however, that the higher expression of WT1 in triple-negative cancers was not due to increased expression in the vascular endothelium.

The sub-analysis of WT1 expression in endothelial cells versus histopathological grade clearly shows that as breast carcinomas become undifferentiated vascular WT1 expression decreases. Grade III carcinomas are associated with poorer clinical outcomes,^52^ suggesting a protective role for endothelial WT1 expression and that perturbations in vascular WT1 expression within the cancers may be crucial. A limitation of the present study is the lack of meaningful clinical outcome data. In future investigations, combining a larger, appropriately powered, patient cohort, with key clinical outcomes—such as disease-free survival and all-cause mortality—would strengthen the ability to draw firm conclusions.

Previous work from our group demonstrated that WT1 was higher in ER-positive than ER-negative tumours.^30^ This contrasts with the results reported here but it should be noted that Artibani et al. measured mRNA expression, whereas the immunohistological approach used in the current study detects the WT1 protein. Furthermore, ER positivity falls with increased grade, so (since WT1 expression dropped as grade increased) a positive correlation of ER status with WT1 expression is to be expected. However, ER-positive and ER-negative breast cancers have been thought to represent distinct disease entities,^37,53^ and ER-negative breast cancers have poorer clinical outcomes than ER-positive tumours.^34^ In this study, ER-positive tumours show greater vascularisation and WT1 endothelial expression compared with ER-negative cancers. Whilst no difference was detected in global WT1 expression between ER-positive and ER-negative tumours, ER-positive tumours exhibited an expression profile markedly different from the matched-control tissue, with increased cellular and vascular WT1 expression. This is mechanistically very interesting as WT1 has been shown to interact with and modulate ER-alpha in vitro.^14^ These data therefore have implications for the development of any future therapeutic agents. For example, the approach for targeting the WT1-peptide vaccine in ER-negative tumours may need to differ significantly compared to the approach taken with tumours which are ER-positive.

Many of the molecular features defining the human breast cancer sub-types have been shown, by DNA microarrays analysis, to be conserved in murine models.^54^ For example, 100% of *C3(1)-Tag* transgenic mice develop breast cancers and produce a “mixed” phenotype, with tumours expressing luminal, basal and mesenchymal genes akin to those seen in basal-like and luminal B human cancers.^55^ Our analyses demonstrated marked histological differences between tumours and control tissues from this model. Interestingly, the *C3(1)/Tag* tumours demonstrated an increase in Wt1^+^ cells, CD31^+^ cells, Wt1/CD31 co-localisation, total vessels and proportion of vessels that are Wt1^+^, compared to healthy control tissue. This is consistent with the WT1 upregulation identified in the human breast cancer samples versus matched controls. Moreover, WT1 was expressed in the healthy control tissue in both species. The observation that the TDLU are WT1-positive both in the tumours and in healthy controls of both mouse and human is consistent with the demonstration that alterations (knock-down or over-expression) in WT1 expression disrupt the epithelial–mesenchymal balance.^30^ Such a dual role is not unexpected given WT1 regulates both epithelial to mesenchymal transition (EMT) and mesenchymal to epithelial transition (MET) during development.^2^ These studies are particularly pertinent given this murine model mimics the proliferation signatures seen in the basal-like human tumours, which are known to be associated with poorer outcomes in younger women.^53^

In conclusion, the data presented in this study demonstrate that the *WT1* gene is expressed in the endothelial (and other) cells of angiogenic tissues. However, whilst WT1 expression is increased in breast cancers, this increase cannot be attributed solely to increased expression in the endothelium. Comparison with control tissues, and stratification of the tumours, suggests that an improved understanding of the role of WT1 in the healthy breast and in breast cancer is required to aid the development of therapies targeting WT1 in this condition. These results emphasise the need for further mechanistic investigations into the influence of WT1 on physiological and pathophysiological angiogenesis. The similarities seen between the human and murine samples support the use of the *C3(1)/Tag* murine tumour model in addressing the role of WT1 in breast cancer.

## Electronic supplementary material

Supplementary Information

## Data Availability

The datasets generated during and/or analysed during the current study are available from the corresponding author on reasonable request
