# Supplementary material for: WT1 expression in vessels varies with histopathological grade in tumour-bearing and control tissue from patients with breast cancer
Source: Br J Cancer. 2018 Oct 30;119(12):1508–17. doi: 10.1038/s41416-018-0317-1 (PMC6288121; doi:10.1038/s41416-018-0317-1)
Supplement: Supplementary file 1 — Supplementary Information [file 41416_2018_317_MOESM1_ESM.docx]

### **SUPPLEMENTARY INFORMATION**

### **Preclinical murine model of breast carcinogenesis**

Tumour formation in the *C3(1)/Tag* system is induced by the expression of the simian virus 40 (SV40) large tumour antigen (Tag) which fuses to and inactivates tumour suppressors *p53* and *Rb* via a rat prostatic steroid binding protein C3(1) (Holzer et al., 2003). The *C3(1)* gene is expressed in both the prostate and mammary glands, and since the SV40 Tag is regulated by the C3(1) promoter, its expression is targeted to the epithelium of both organs, driving the development of prostate tumours in males and mammary tumours in female mice (Maroulakou et al., 1994). Tumours develop without the need for hormone treatment or pregnancy so the model is a more accurate representation of human disease (Green et al., 2000). By 8 weeks of age, the mice demonstrate atypia of the mammary ductal epithelium, which progresses to intra-epithelial neoplasia analogous to ductal carcinoma *in situ*, culminating in 100% of animals developing invasive carcinoma by 16 weeks of age (Green et al., 2000).

**
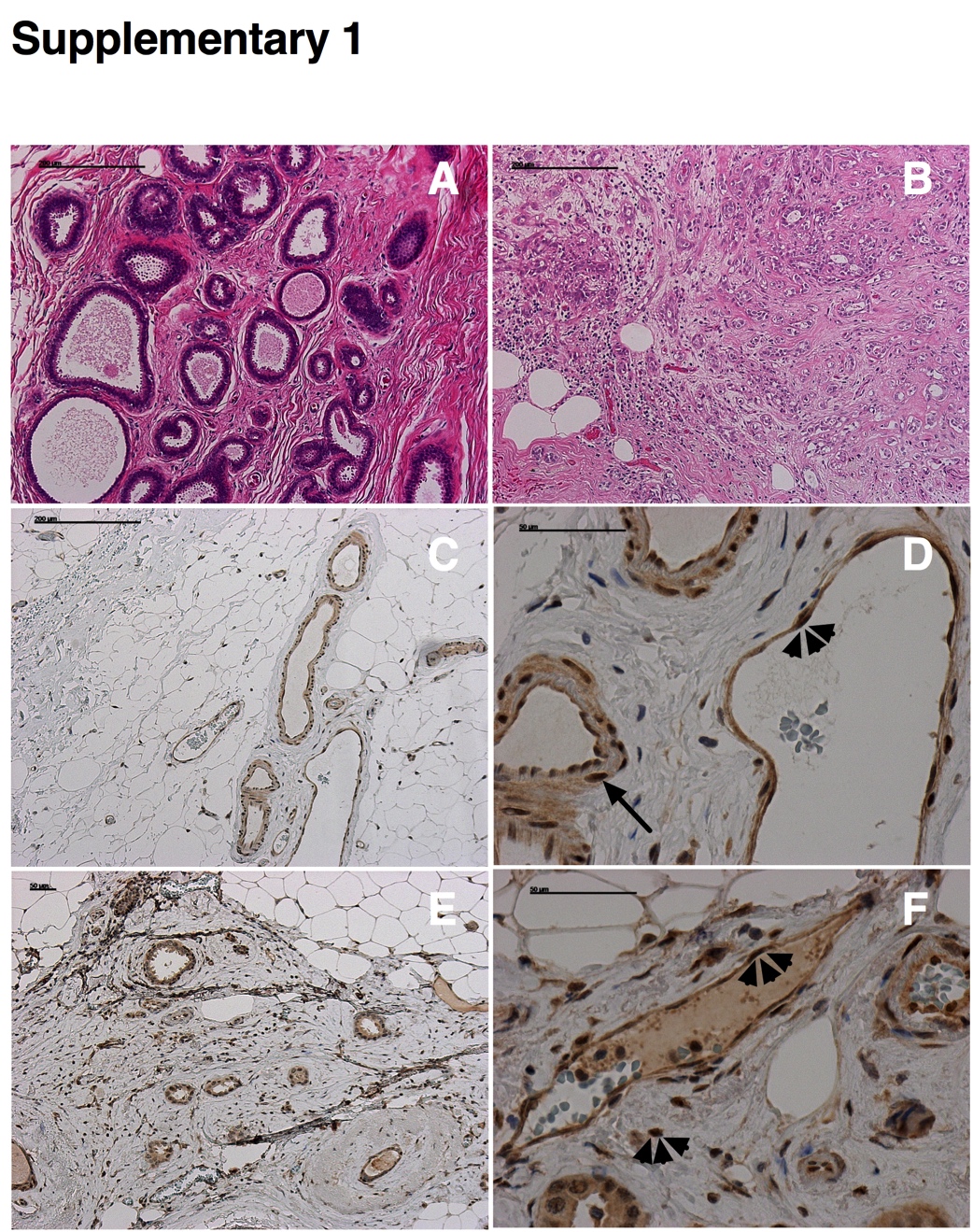
**

**Figure S1. Immunohistochemical analysis of healthy human breast tissue versus Grade II breast cancer (representative images of n=4 Grade II tumours versus matched controls). A**. Benign breast tissue illustrated by haematoxylin and eosin stain (H&E – note the terminal duct lobular unit (TDLU) in deep purple). **B.** Breast tissue widely infiltrated by ductal adenocarcinoma (Grade II) on H&E staining. **C.** WT1 stain (brown) of healthy human breast tissue depicting WT1^+^ capillaries and arteries **D**. Higher magnification reveals WT1^+^ endothelial cells in the capillaries and arteries (arrowhead), alongside WT1^+^smooth muscle cells in the arterial walls (black arrow). **E.** Widely infiltrated ductal adenocarcinoma stained using WT1 DAB. **F.** Higher magnification; WT1^+^ is present in the tumour stromal and endothelial cells (as indicated by arrowheads). **Key**: Scale bars in **A-C** represent 200 µm and **D-F** 50 µm.


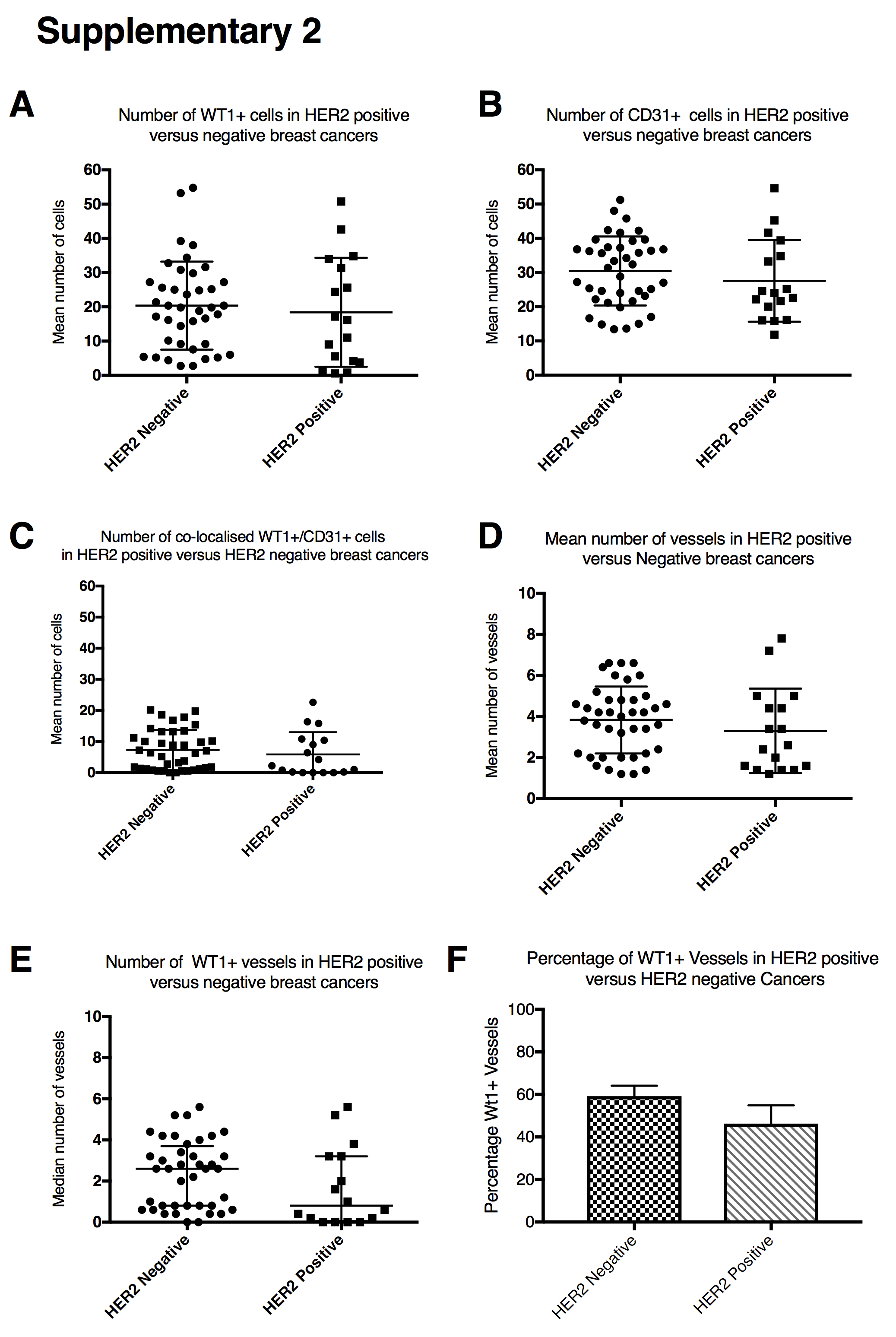


**Figure S2. The relationship between WT1 expression and HER2 receptor status in 57 human breast cancers.** No difference was detected between HER2 negative and HER2 positive breast cancers regarding the number of WT1^+^ cells **(A)**; number of CD31^+^ cells **(B)**; cells co-expressing WT1/CD31 **(C);** the total number of vessels (**D**); and the number of WT1^+^ vessels **(E)**. The percentage of WT1^+^ vessels in HER2 positive versus HER2 negative tumours also exhibited no significant difference (**F**) (where n=40 HER2 negative tumours, 17 HER2 positive tumours, **A/B/C/D** data are mean ± standard deviation **and E** data are median ± interquartile range)


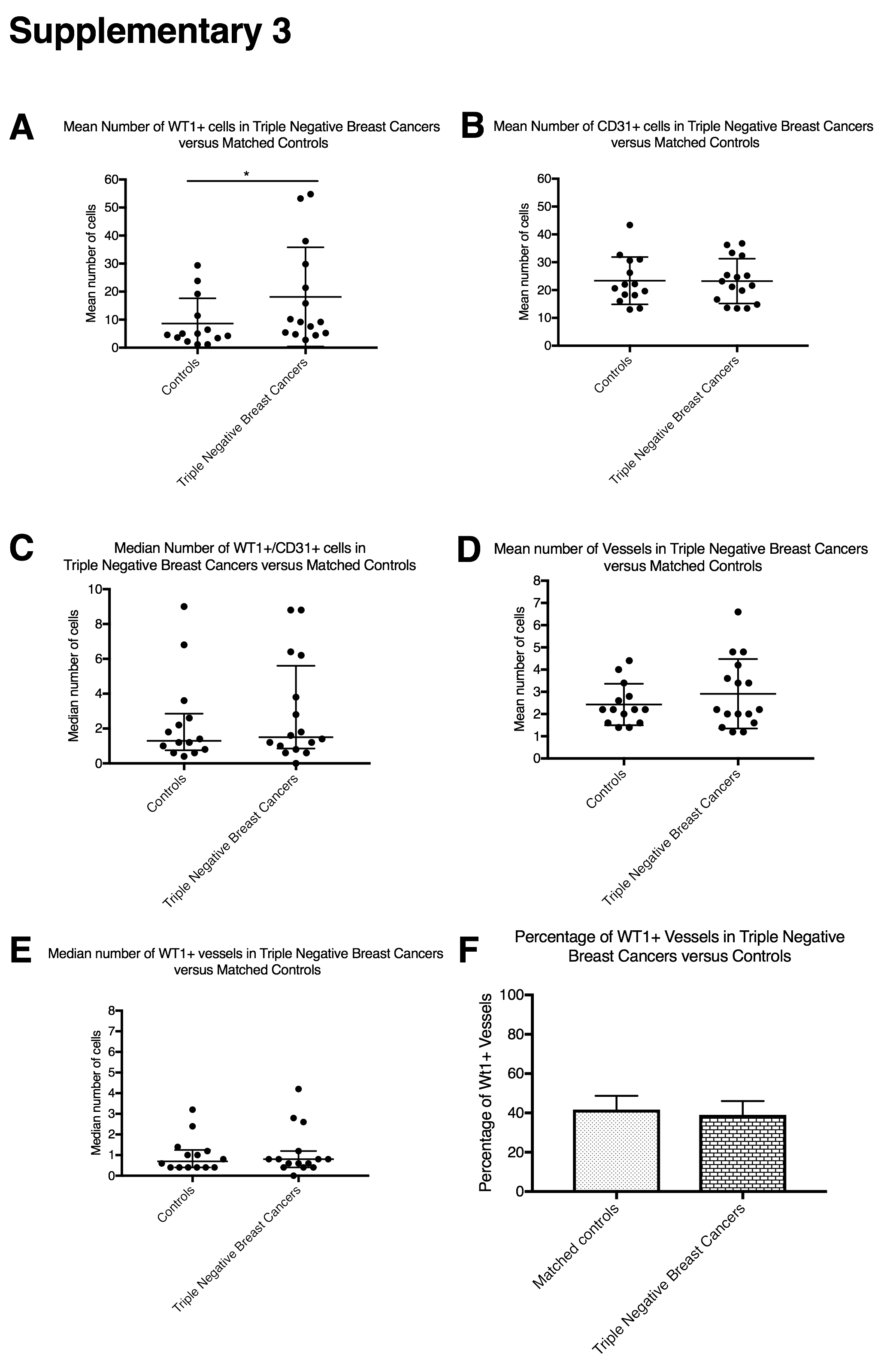


**Figure S3. The relationship between WT1 expression and Triple Negative human breast cancers versus matched controls.** The number of WT1+ cells was increased in the Triple negative breast cancers versus matched controls (**A**), but there was no difference in the number of CD31+ cells **(B)**, WT1/CD31 co-localisation **(C)**, number of vessels **(D)**, number of WT1+ vessels **(E)**, or the percentage of WT1+ vessels **(F)** (where n = 15 triple negative breast cancers, n = 14 matched controls, **A/B/D/F** data are mean ± standard deviation where ★ p < 0.05 by unpaired Student’s t test, and **C/E** are median ±interquartile range).


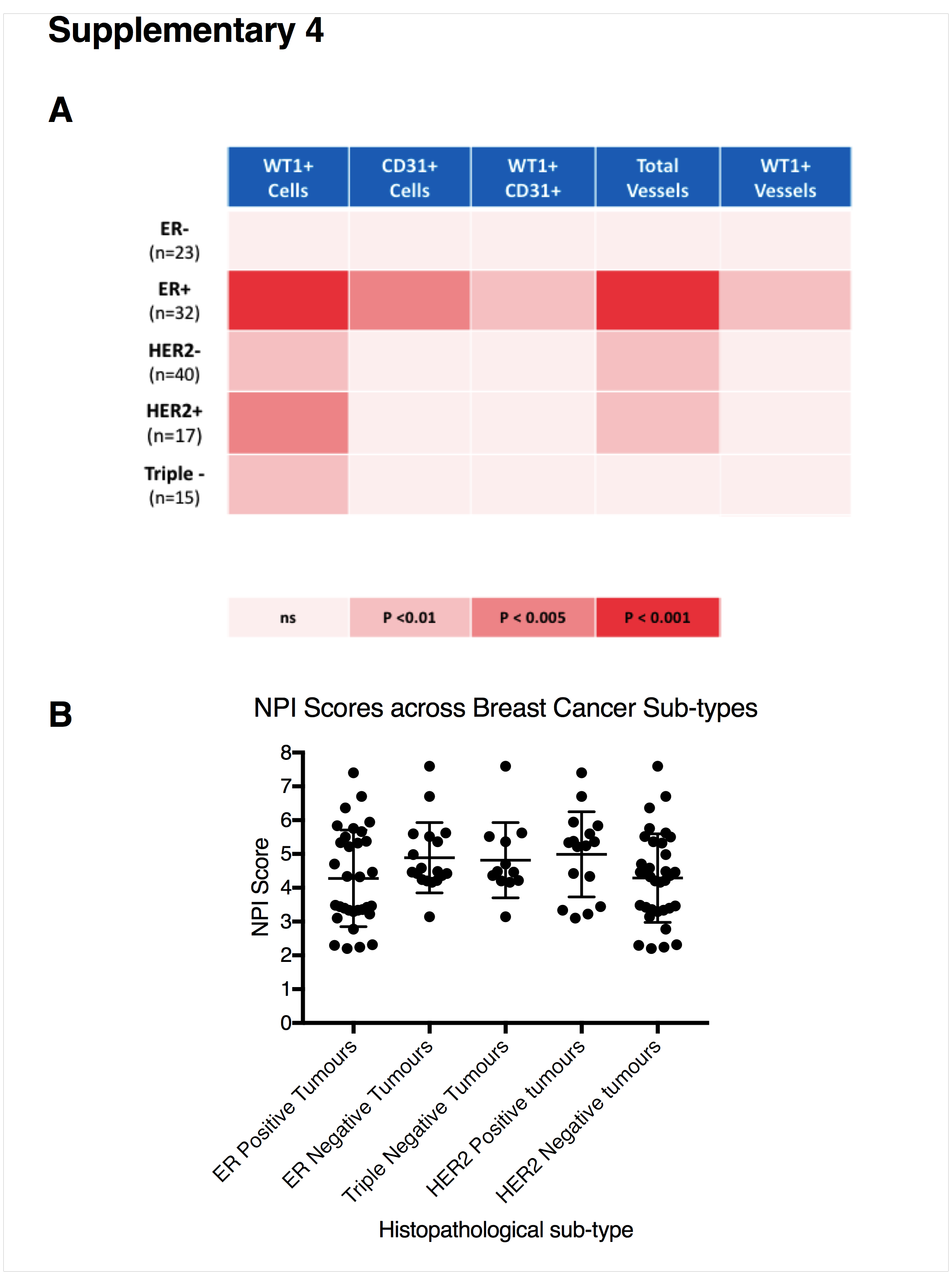


**Figure S4. The relationship between WT1 expression in a variety of cancer sub-types and matched control tissue. A.** Heat map depicting the variation in expression of WT1 across a sub-set of breast carcinomas, relative to matched control tissue. Statistically significant results are represented by the degree of colour change within the panels. **B.** The in NPI score across breast cancer sub-types shows no significant difference, with the exception of Grade I tumours


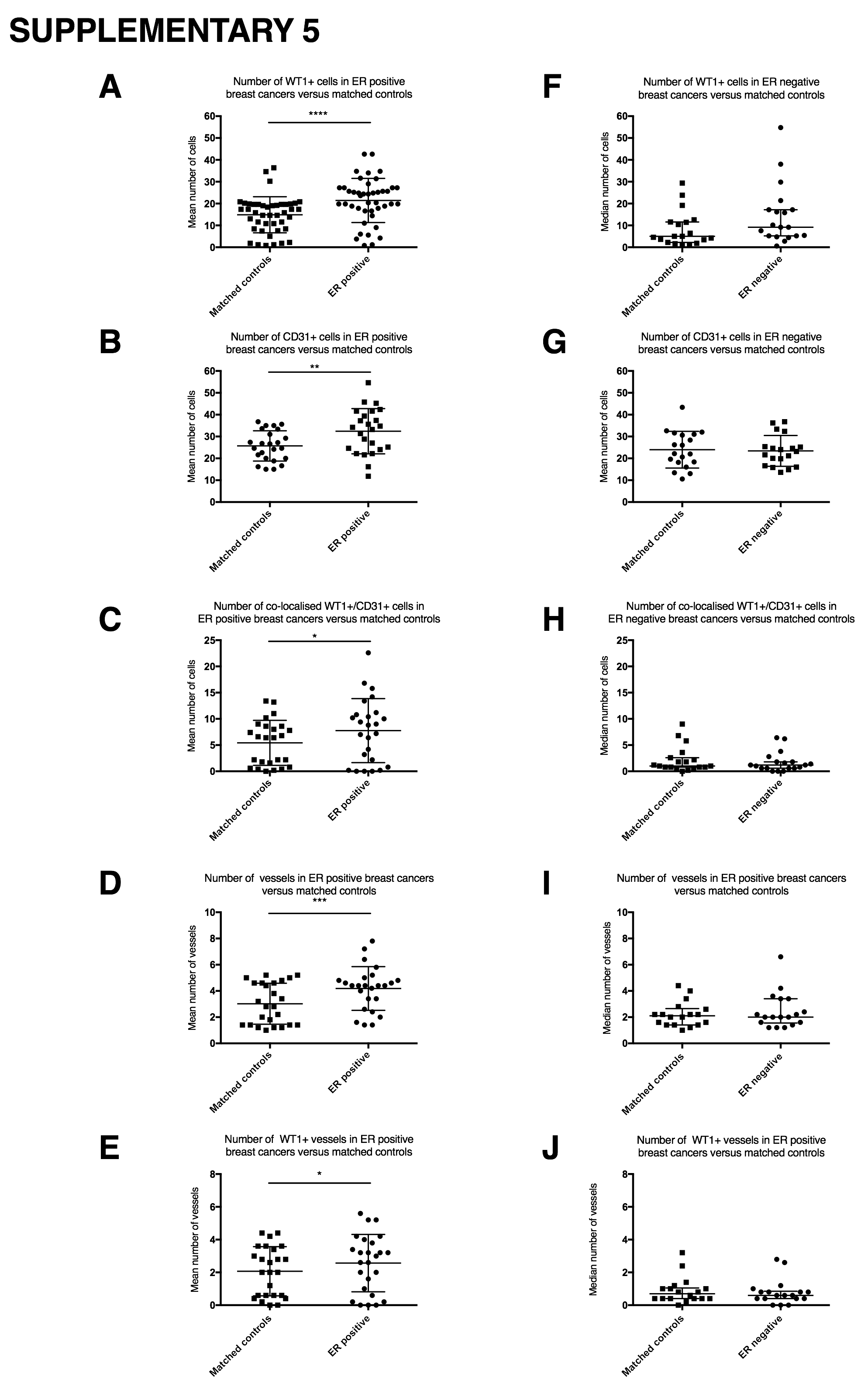


**Figure S5. The relationship between WT1 expression and ER positive and negative human breast cancers versus matched controls.** The number of WT1+ cells, CD31+ cells, WT1/CD31 co-localisation, number of vessels, and number of WT1+ vessels were all increased in ER positive breast cancers versus matched controls (**A-E**). However, no difference was detected in the same analysis across ER negative breast cancers versus matched control tissue (**F-J**) (where n = 43 ER positive cancers with matched controls and n = 19 ER negative with matched controls, **A/B/C/D/E/G** data are mean ± standard deviation where ★ p < 0.05, ★★ p < 0.005, ★★★ p < 0.0005, ★★★★ p < 0.0001 by unpaired Student’s t test, and **F/H/I/J** are median ±interquartile range).
